# Supplementary material for: Application of Solvent Evaporation to Generate Supersaturated Lipid-Based Formulations: Investigation of Drug Load and Formulation Quality
Source: Pharmaceutics. 2025 May 27;17(6):702. doi: 10.3390/pharmaceutics17060702 (PMC12196494; doi:10.3390/pharmaceutics17060702)
Supplement: Supplementary file 1 [file pharmaceutics-17-00702-s001.zip › pharmaceutics-3558112-supplementary.pdf]

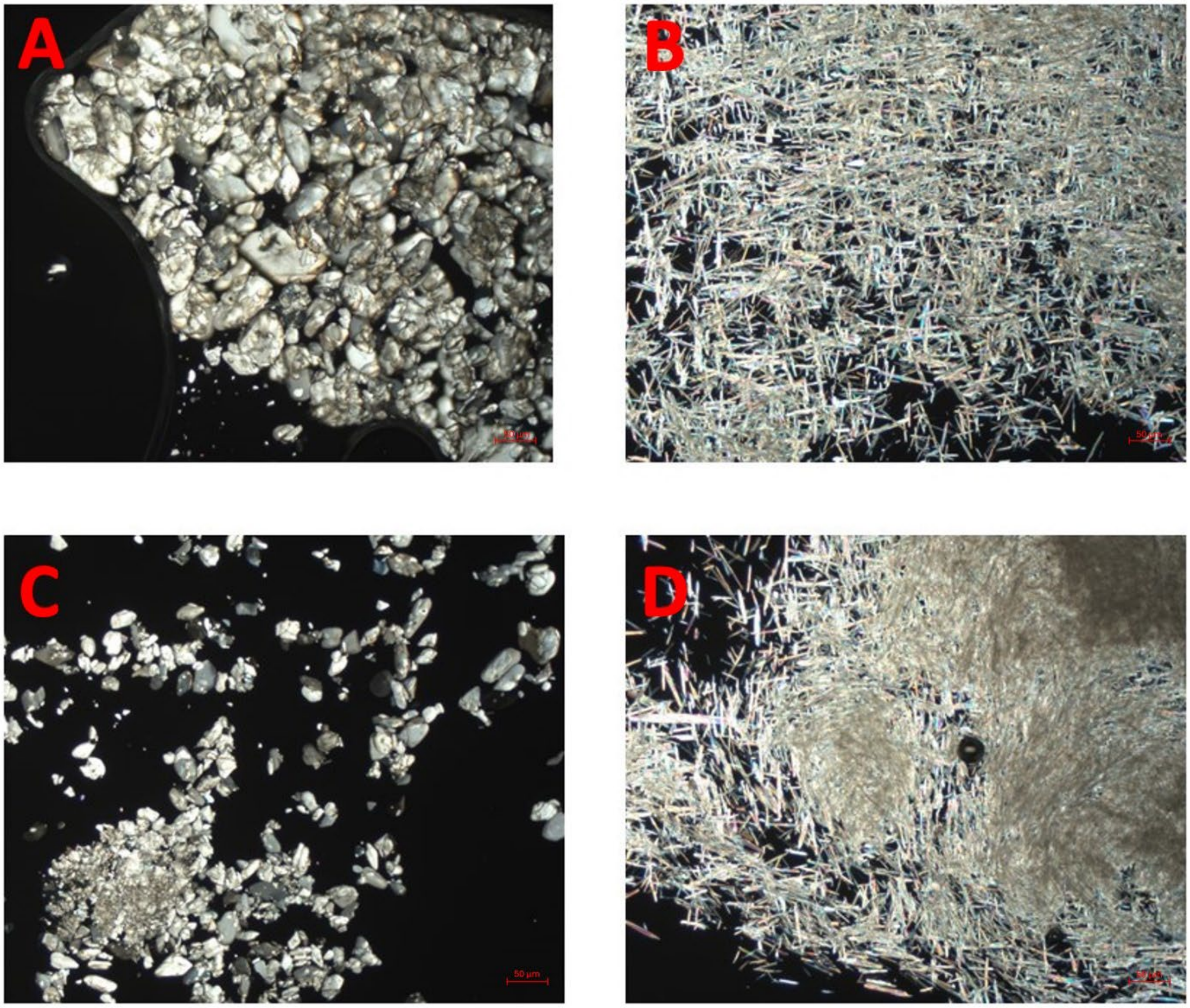

Figure S1: Celecoxib- and fenofibrate-powder in lipid excipients under polarized light. (A) Fenofibrate in MCT. (B) Celecoxib in MCT. (C) Fenofibrate in LCM. (D) Celecoxib in LCM.
